# Naoxueshu Oral Liquid Attenuates Early Radiation‐Induced Brain Injury in Rats

**DOI:** 10.1002/brb3.71801

**Published:** 2026-10-14

**Authors:** Fuguang Li, Xiaolong Qu, Kuiyuan Li, Guosong Li, Shifang Zhou, Min Zhao, Chunyu Zhang, Lei Hui, Wenchao Li

**Affiliations:** ^1^ Department of Neurosurgery The First Affiliated Hospital of Henan Medical University; Henan medical Key Laboratory of Neurology, Henan Joint International Laboratory of Neurorestoratology for Senile Dementia, Henan Engineering Technology Research Center of Neurorestoratology ,Henan Engineering Laboratory of Neurorestoratology,Henan Key Laboratory of Neurorestoratology and Protein modification, First Affiliated Hospital of Henan Medical University, Xinxiang, Henan, 453100, China. Xinxiang City China

**Keywords:** brain edema, brain injuries, cognitive impairment, Naoxueshu Oral Liquid, radiation injuries

## Abstract

**Objective:**

To investigate the effect of Naoxueshu Oral Liquid on rats with radiation‐induced brain injury.

**Methods:**

A total of 108 male Sprague–Dawley rats were randomly assigned to a control group, an irradiated + normal saline group (RBI+NS), and an irradiated + Naoxueshu Oral Liquid group (RBI+NXS). A radiation‐induced brain injury model was established by single‐dose whole‐brain high‐energy X‐ray irradiation (30 Gy). After irradiation, rats received Naoxueshu Oral Liquid (0.5 mL/100 g/day, gavage) or normal saline for 14 days. Body weight was monitored, cerebral edema was assessed by brain tissue water content on Days 3, 7, and 14, histopathology was evaluated by hematoxylin–eosin (H&E) staining, and Morris water maze training was conducted from Day 8 to Day 13 post‐irradiation, followed by the probe trial on Day 14 post‐irradiation.

**Results:**

Body weight differed significantly among groups after irradiation (Day 3/6/9/12: all *p* < 0.01). Specifically, at Day 12, body weight was 328.17±20.10 g (control), 258.50±18.87 g (RBI+NS), and 302.83 ± 10.87 g (RBI+NXS); RBI+NXS was higher than RBI+NS (*p* < 0.01), while control versus RBI+NS remained different (*p* < 0.01). H&E staining showed widened perineuronal and perivascular spaces after irradiation, which were attenuated by Naoxueshu. In the Morris water maze, RBI+NS rats exhibited prolonged latency and fewer platform crossings; these impairments were improved after Naoxueshu treatment.

**Conclusion:**

Naoxueshu Oral Liquid reduced cerebral edema and improved Morris water maze performance in rats after whole‐brain irradiation. Because behavioral outcomes were assessed only using the Morris water maze, the cognitive‐related findings should be interpreted cautiously.

## Graphical abstract

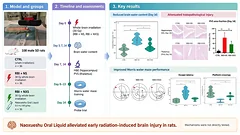

## 1 Introduction

Radiotherapy is a core treatment modality for head and neck malignancies (such as nasopharyngeal carcinoma, glioma, etc.), playing a critical role in improving local tumor control rates and patient survival rates (Liu et al. 2025; Rübe et al. 2023). However, while radiation effectively kills tumor cells, it often causes irreversible damage to normal brain tissue, leading to the development of radiation‐induced brain injury (RBI) (Sun et al. 2025; Tian et al. 2025). RBI can manifest gradually from several days to years after radiotherapy, with clinical symptoms including progressive cognitive decline, motor dysfunction, epileptic seizures, and cerebral edema, among others. These symptoms not only severely reduce patients' quality of life but may even lead to treatment interruption, making RBI a major limiting factor in the efficacy of radiotherapy (Wu et al. 2024). Current clinical management of RBI primarily relies on symptomatic treatments such as corticosteroids and neurotrophic agents (Greene‐Schloesser et al. 2012). However, long‐term use of these interventions can lead to side effects such as infections and osteoporosis, and their efficacy in improving cognitive function remains limited (Wang et al. 2025). Therefore, exploring safe and effective prevention and treatment strategies is of significant clinical importance. Recent basic and translational studies have shown that RBI is a multifactorial process involving neuroinflammation, oxidative stress, blood–brain barrier dysfunction, microvascular injury, and hippocampal damage rather than a single dominant lesion.

Neuroinflammation is one of the major components of this process. Radiation can induce abnormal microglial activation and promote the release of pro‐inflammatory mediators such as IL‐1β and TNF‐α, thereby aggravating neuronal and vascular injury (Lumniczky et al. 2017; Schnegg et al. 2013; Xiong et al. 2025). In parallel, radiation‐induced oxidative stress disrupts lipids, proteins, and nucleic acids and contributes to neuronal apoptosis (Turnquist et al. 2020). Blood–brain barrier disruption is also important, because reduced expression of tight junction proteins such as ZO‐1 may increase vascular permeability and promote cerebral edema (Luo et al. 2024; Wang et al. 2025). In addition, the hippocampus is highly sensitive to irradiation, and hippocampal injury is closely associated with learning and memory impairment (Chen et al. 2025; Pazzaglia et al. 2021). Taken together, these features indicate that RBI is not driven by a single pathway, and this complexity provides a rationale for exploring interventions with multimodal regulatory potential.

Traditional Chinese Medicine (TCM) has historically emphasized integrated regulation in complex injury states. From a contemporary biomedical perspective, this conceptual framework is relevant to RBI because the disorder involves interacting inflammatory, vascular, edematous, and neuronal mechanisms rather than a single isolated lesion. According to traditional theory, brain injury is often associated with blood stasis obstructing the collaterals and insufficient nourishment of the brain, and treatment is therefore directed toward promoting circulation, unblocking the collaterals, replenishing qi, and restoring brain function (Ding 2017; Ding et al. 2023). This multi‐component and multi‐target therapeutic concept has also been increasingly discussed in modern studies of TCM‐based neurovascular intervention, particularly in relation to the regulation of neuroinflammation, oxidative stress, and neurovascular unit integrity (Lv et al. 2024; Wu et al. 2024).

Naoxueshu Oral Liquid, a commonly used clinical TCM formulation developed based on these principles, contains key ingredients such as *Astragalus membranaceus* and *Ligusticum chuanxiong*. Previous studies have suggested that its pharmacological profile may be relevant to several pathological components of RBI. For example, active components in *Astragalus membranaceus* can alleviate neuroinflammation by inhibiting inflammatory signaling pathways such as NF‐κB and p38 MAPK (Sun and Wang 2024) and reduce neuronal apoptosis by activating the PI3K/Akt pathway (Zhang et al. 2025). Recent evidence has further highlighted the neuroprotective potential of *Astragalus*‐related constituents in neurological disorders, including anti‐neuroinflammatory and pro‐survival signaling effects (Shi and Ma 2024). Ligustrazine from *Ligusticum chuanxiong* can enhance antioxidant capacity by increasing superoxide dismutase (SOD) activity, while also improving cerebral microcirculation and protecting blood–brain barrier integrity (Li and Lan 2011; Luo et al. 2015). In addition, recent experimental studies have shown that tetramethylpyrazine can preserve blood–brain barrier integrity, attenuate brain edema, and suppress inflammatory cytokine expression in models of cerebral injury (Jin et al. 2021). Furthermore, clinical studies have demonstrated that Naoxueshu Oral Liquid effectively reduces cerebral edema and improves neurological deficits in conditions such as cerebral hemorrhage and cerebral infarction (Cao 2019; Wang et al. 2024). More recent clinical and mechanistic studies have also suggested that Naoxueshu may promote hematoma absorption, relieve cerebral edema, improve neurological function, and modulate inflammation‐related pathways in hemorrhagic brain injury (Li et al. 2023; Song et al. 2022). Accordingly, the rationale for evaluating Naoxueshu in RBI lies in the conceptual overlap between its reported multi‐component pharmacological properties and the multifactorial pathophysiology of radiation‐induced brain injury. However, its specific role and underlying mechanisms in RBI remain unclear. Against this background, the present study established a rat model of RBI to evaluate the effects of Naoxueshu Oral Liquid on body weight, brain water content, histopathological changes, and Morris water maze performance. The aim was to assess whether Naoxueshu could mitigate early injury manifestations in an acute high‐dose RBI model, while interpreting mechanistic implications cautiously within the limits of the available dataset.

## 2 Materials and Methods

### 2.1 Materials

#### 2.1.1 Experimental Animals

SPF‐grade 10‐week‐old male Sprague–Dawley rats (body weight: 180–200 g) (Certificate No.: 4100000001370) were purchased from Henan Sikebisi Biotechnology Co., Ltd. All rats were housed in the standard animal facility of the Life Science Research Center at The First Affiliated Hospital of Xinxiang Medical University, under controlled conditions: temperature 25 ± 2°C, a 12‐h light/dark cycle, with free access to food and water. This study was conducted in compliance with animal ethics guidelines and was approved by the Ethics Committee of The First Affiliated Hospital of Xinxiang Medical University (Approval No.: EC‐023‐217). Only male rats were used to minimize potential variability related to the estrous cycle and sex hormone–dependent neurobehavioral and inflammatory responses, which could confound outcome measures such as cerebral edema and Morris water maze performance.

#### 2.1.2 Drugs and Equipment

Naoxueshu Oral Liquid (10 mL × 10 vials/box; National Medicine Permit No.: Z20070059) was purchased from Shandong Wohua Pharmaceutical Co., Ltd. The product complied with the National Drug Standard (New Drug Confirmation Standard), Volume 71, WS3‐111(Z‐021)‐2005(Z). Its primary ingredients include *Astragalus membranaceus* (Huangqi), *Hirudo* (Shuizhi), *Acorus tatarinowii* (Shichangpu), *Cyathula officinalis* (Niuxi), *Cortex moutan* (Mudanpi), *Rheum palmatum* (Dahuang), and *Ligusticum chuanxiong* (Chuanxiong) (Wang et al. 2024). The preparation method was as follows: Volatile oils were first extracted from *Acorus tatarinowii* and *Cortex moutan* via distillation, and the resulting aqueous solution was collected separately. *Astragalus membranaceus*, Cyathula officinalis, *Ligusticum chuanxiong*, and *Rheum palmatum* were decocted twice with water (first with 6–10 times and second with 4–8 times the amount of water), each time for 2 h. The decoctions were combined, filtered, and mixed with the previously collected aqueous solution from distillation. The mixture was concentrated to a relative density of 1.08–1.10 (80°C –85°C). A suitable amount of ethanol was added, followed by mixing and standing. The supernatant was collected, and the ethanol was recovered by concentrating the solution to a relative density of 1.20–1.25 (70°C –80°C). Hirudo was decocted twice separately; the decoctions were combined, filtered, and concentrated to a relative density of 1.04–1.06 (70°C –80°C). This was then combined with the previous concentrate, an appropriate amount of water was added, and the pH was adjusted. The solution was heated to boiling, allowed to stand, and filtered. The volatile oils from *Acorus tatarinowii* and Cortex moutan (pre‐mixed with Tween‐80 equivalent to the volume of the oils) were added to the filtrate. The mixture was homogenized, brought to a total volume of 1000 mL with water, thoroughly stirred, packaged, and sterilized (Shandong Wolwa Pharmaceutical Technology Co., Ltd 2008). The Naoxueshu dosing regimen used in this study was determined based on interspecies dose conversion in translational pharmacology: the rat‐equivalent dose is commonly ∼6‐ to 10‐fold the adult dose on a mg/kg (or mL/kg) basis. Given that the routine adult daily dose is approximately 0.5–0.6 mL/kg, the rat dose was set at 0.5 mL/100 g (i.e., 5 mL/kg) for intragastric administration. Vital Beam Medical Linear Accelerator was provided by The First Affiliated Hospital of Xinxiang Medical University. The irradiation dose rate was set at 800 cGy/min. A single‐dose irradiation paradigm was adopted to establish a stable and reproducible acute brain injury phenotype within a practical experimental timeframe for evaluating short‐term therapeutic responses. This experimental design was intended to capture early radiation‐induced injury within a short observation window rather than to directly reproduce routine clinical fractionated radiotherapy. Morris Water Maze System was provided by Jiangsu Sciens Biotechnology Co., Ltd. The supporting data analysis software was the company's dedicated Morris Water Maze behavioral analysis system. The equipment was configured and used by the Life Science Research Center of The First Affiliated Hospital of Xinxiang Medical University. Hematoxylin and Eosin (H&E) Staining Kit was purchased from Beijing Solarbio Science & Technology Co., Ltd.

### 2.2 Methods

#### 2.2.1 Experimental Grouping

A total of 108 rats were randomly divided into three groups: Control group (*n* = 36): Underwent anesthesia and positioning but received no radiation exposure. To control for the potential effects of intragastric gavage, rats in this group received normal saline by gavage (0.5 mL/100 g body weight) once daily for 14 consecutive days, using the same schedule and handling procedures as the irradiated groups. Radiation + Normal Saline group (R+NS, *n* = 36): Underwent single‐dose whole‐brain irradiation (30 Gy). This group received intragastric administration of normal saline (0.5 mL/100 g body weight) daily for 14 consecutive days post‐irradiation. Radiation + Naoxueshu Oral Liquid group (R+NXL, *n* = 36): Underwent single‐dose whole‐brain irradiation (30 Gy). This group received intragastric administration of Naoxueshu Oral Liquid (0.5 mL/100 g body weight) daily for 14 consecutive days post‐irradiation. Following 1 week of adaptive feeding, all rats were anesthetized via intraperitoneal injection of 1% sodium pentobarbital (4 mg/100 g body weight). Successfully anesthetized animals were placed in a prone position on the radiotherapy platform. The radiation field was precisely defined using lead plates to expose only the brain area while shielding the eyes and neck. Specifically, lead blocks were mounted on an overhead support frame above the animal's head to collimate the irradiation field; by adjusting the position/angle of the lead blocks, the exposed field was restricted to the predefined cranial target region. This shielding assembly corresponds to the overhead bracket visible in Figure 1, and the resulting illuminated/irradiated area is the head region shown in the figure. Whole‐brain irradiation was performed using a Vital Beam medical linear accelerator, delivering a single dose of 30 Gy at a source‐to‐skin distance (SSD) of approximately 100 cm (as shown in Figure 1). Animals in the Control group underwent identical anesthesia and positioning procedures but did not receive radiation exposure. All groups underwent identical gavage handling and volume administration as described above. To ensure sufficient animals for multiple planned terminal endpoints at different time points, a pre‐specified allocation scheme was used. Each group started with *n* = 36 rats and animals were randomly assigned to each endpoint/time point (6 rats per group per time point): brain water content (wet/dry weight) was assessed on Days 3, 7, and 14 (6 rats/group at each time point); H&E was performed on Days 7 and 14 (6 rats/group at each time point). For the Morris water maze experiment, a separate cohort of 6 rats per group was identified on Day 8 post‐irradiation. Therefore, the effective sample size for each analysis (including MWM) was *n* = 6 rats per group, consistent with the planned terminal sampling design, providing six independent animals for each endpoint (Liu et al. 2023; Xiong et al. 2025). No a priori power calculation was performed before the initiation of this exploratory animal study. The sample size of *n* = 6 per group for each endpoint was determined according to the predefined endpoint‐specific experimental design, including terminal brain water content assessment, histopathological evaluation, and Morris water maze analysis, with allocation of independent biological replicates for each assessment. This single‐dose whole‐brain irradiation protocol was designed to model acute radiation‐induced brain injury and to enable detection of early endpoints (cerebral edema, histopathological injury, and early cognitive impairment) within 14 days. Because this acute high‐dose protocol does not reproduce the dose fractionation or temporal evolution of clinical radiotherapy‐associated brain injury, the present model should be interpreted as an experimental paradigm for early radiation‐induced changes rather than a direct simulation of routine clinical fractionated radiotherapy. Accordingly, this paradigm was used specifically to investigate early experimental RBI and short‐term treatment‐associated changes within the 14‐day observation window.

**FIGURE 1:**
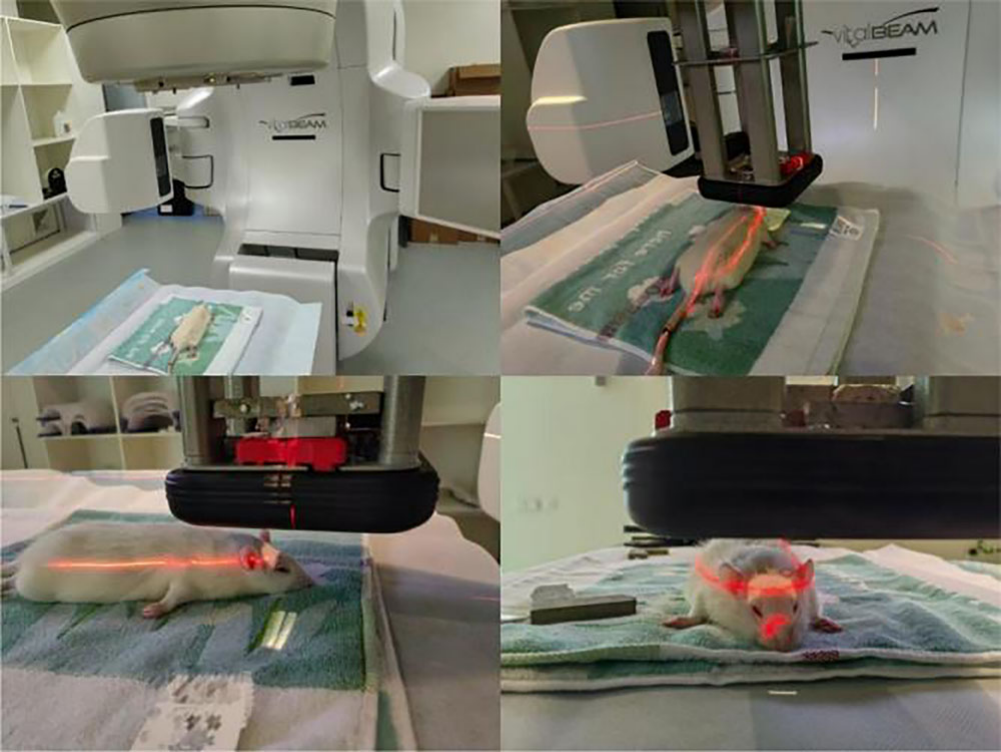
Establishment of a rat model of radiation‐induced brain injury (RBI). Representative photographs showing whole‐brain irradiation delivered by a medical linear accelerator (Vital Beam). Male Sprague–Dawley rats were anesthetized and positioned prone on the radiotherapy platform, and a single dose of 30 Gy whole‐brain irradiation was administered (dose rate: 800 cGy/min; source‐to‐skin distance: ∼100 cm, as described in Section 2.2). The irradiation field was collimated to the brain using lead shielding to protect non‐target regions including the eyes and neck (see Section 2.2).

#### 2.2.2 Morris Water Maze Test

For the Morris water maze experiment, 6 rats in each group were identified on Day 8 post‐irradiation and assigned to the behavioral testing cohort. Acquisition training was conducted from Day 8 to Day 13 post‐irradiation. A removable escape platform was placed at a fixed location within the target quadrant. For the first 4 days of training, the platform was maintained 1 cm above the water surface (visible platform). The initial visible‐platform phase was used to familiarize the rats with the water maze procedure and establish the platform‐escape rule before transition to hidden‐platform spatial training. The following hidden‐platform phase was used to assess spatial learning‐ and memory‐related task performance, according to modified Morris water maze procedures described previously (Morris 1984). For the subsequent 2 days, the platform was submerged 1 cm below the water surface (hidden platform), requiring the animals to locate the fixed platform using spatial cues. During each training session, rats were placed into the water facing the pool wall from one of four different starting quadrants (randomly assigned for each trial) and were allowed a maximum of 60 s to locate the platform. If a rat failed to find the platform within 60 s, it was gently guided to the platform and allowed to remain on it for 15 s. The escape latency (time taken to find the platform) within the 60‐second period was recorded for each trial. On Day 14 post‐irradiation, the platform was removed. Each rat was introduced into the pool facing the wall from the quadrant opposite to the former target quadrant and allowed to swim freely for 60 s. The following parameters were recorded for each animal: the latency to the first crossing of the former platform location and the number of crossings over the exact former platform location.

#### 2.2.3 Body Weight Measurement

Starting from the first day after irradiation, 6 rats were randomly selected from each group and ear‐tagged, and the same animals were weighed daily for body weight measurement, and the weight of each rat was recorded. All measurements were performed at a consistent time window each day.

#### 2.2.4 Brain Water Content

Brain water content was evaluated using the wet‐dry method at 3, 7, and 14 days after modeling. At each time point, 6 rats were randomly selected from each group. After anesthesia with an intraperitoneal injection of 1% sodium pentobarbital (4 mg/100 g), the rats were decapitated, and the brains were rapidly harvested. The wet weight of each brain was immediately measured. The brain tissues were then dried at 80°C for 72 h and reweighed to obtain the dry weight. The brain water content was calculated as follows: [(wet weight − dry weight) / wet weight] × 100% (Gottschalk et al. 2021).

#### 2.2.5 Histopathological Examination

At 7 and 14 days after modeling, 6 rats were randomly selected from each group at each time point. The rats were anesthetized with an intraperitoneal injection of 1% sodium pentobarbital (4 mg/100 g) and transcardially perfused first with buffered saline followed by 4% paraformaldehyde. The entire brain was carefully removed. Coronal sections of 2 mm thickness were obtained and fixed, followed by dehydration, paraffin embedding, and preparation of continuous coronal sections with a thickness of 5 µm. The sections were routinely stained with hematoxylin and eosin (H&E) after deparaffinization, rehydration, staining, dehydration, and clearing. Finally, the sections were coverslipped and observed under an optical microscope for imaging. Representative images were acquired from the hippocampal dentate gyrus (DG) and the thalamic region on coronal sections at comparable anatomical levels using a consistent imaging protocol. In addition to descriptive histological observations, the perivascular space (PVS) area fraction has also been used as a semiquantitative morphometric indicator of vascular‐related morphological changes. The PVS area fraction refers to the proportion of the perivascular space void area relative to the total vascular area.

#### 2.2.6 Data Processing

Statistical analysis was performed using SPSS 27.0 software, and graphs were generated with Prism 8.0. Measurement data are expressed as mean ± standard deviation ($\bar{x}\pm s$). For all endpoints, the actual sample size analyzed was *n* = 6 rats per group (as reported in each analysis). Body weight data were analyzed using repeated measures ANOVA. Results of the Morris water maze test were analyzed using one‐way ANOVA for intergroup comparisons. Brain water content data were analyzed using the Kruskal–Wallis test for overall comparisons among the three groups at each time point. If the overall test indicated a significant difference, pairwise comparisons were performed using the Mann–Whitney U test with Bonferroni correction for multiple comparisons. A *p*‐value of less than 0.05 was considered statistically significant.

## 3 Results

### 3.1 Effect of Naoxueshu Oral Liquid on Body Weight in Rats Post‐Irradiation

Changes in body weight across groups at various time points are summarized in Table 1. Compared with the Control group, both the Radiation + Normal Saline (R+NS) group and the Radiation + Naoxueshu Oral Liquid (R+NXL) group showed significantly slower weight gain (*p* < 0.01). Specifically, the R+NS group exhibited weight loss within the first 3 days after irradiation, with body weight decreasing to (228.50 ± 9.71) g. Although weight gradually recovered thereafter, it reached only (258.50 ± 18.87) g by Day 12, representing a 13.1% increase from Day 3. In contrast, the R+NXL group showed no significant decrease in body weight at 3 days post‐irradiation (250.00 ± 9.73 g), and their weight increased steadily over time. By Day 12, body weight in this group reached (302.83 ± 10.87) g, reflecting a 21.1% increase from Day 3. The rate of weight gain in the R+NXL group was significantly faster than that in the R+NS group (Figure 2). Treatment with Naoxueshu Oral Liquid resulted in a significant increase in body weight compared with the R+NS group (*p* < 0.01), and the growth trend approached that of the Control group after 1 week. Rats in the Control group demonstrated weight gain consistent with the standard growth pattern of 10‐week‐old male SPF SD rats under conventional rearing conditions, likely attributable to a stable experimental environment and ad libitum access to food. Repeated measures ANOVA indicated that the overall trends in weight gain differed significantly among the three groups (all *p* < 0.01 at each measured time point). The R+NS group showed the slowest growth, while the R+NXL group exhibited better recovery, gradually approaching the weight profile of the Control group.

**TABLE 1:** Body weight of rats in each group at different time points.

Time (Day)	Control	Irradiation (Saline)	Irradiation (Drug)	*F*‐value	*p*‐value	Key intergroup *p*‐values
0	241.83 ± 5.12	238.00 ± 6.29	245.67 ± 4.41	3.103	0.075	All pairwise comparisons *p* > 0.05
3	264.33 ± 6.89	228.50 ± 9.71	250.00 ± 9.73	24.858	<0.01	Control vs. Irradiated Group (Saline) or (Drug) *p* < 0.01; Irradiated Group (Saline) vs. Irradiated Group (Drug) *p* < 0.01
6	286.67 ± 11.69	237.00 ± 15.32	261.00 ± 14.56	19.032	<0.01	Control vs. Irradiated Group (Saline) or (Drug) *p* <0.01; Irradiated Group (Saline) vs. Irradiated Group (Drug) *p* < 0.01
9	307.61 ± 15.33	243.50 ± 20.17	278.00 ± 11.63	23.904	<0.01	Control vs. Irradiated Group (Saline) or (Drug) *p* < 0.01; Irradiated Group (Saline) vs. Irradiated Group (Drug) *p* < 0.01
12	328.17 ± 20.10	258.50 ± 18.87	302.83 ± 10.87	24.460	<0.01	Control vs. Irradiation + Saline *p* < 0.01; Control vs. Drug Group *p* > 0.05; Irradiation + Saline vs. Irradiation + Drug *p* < 0.01

**FIGURE 2:**
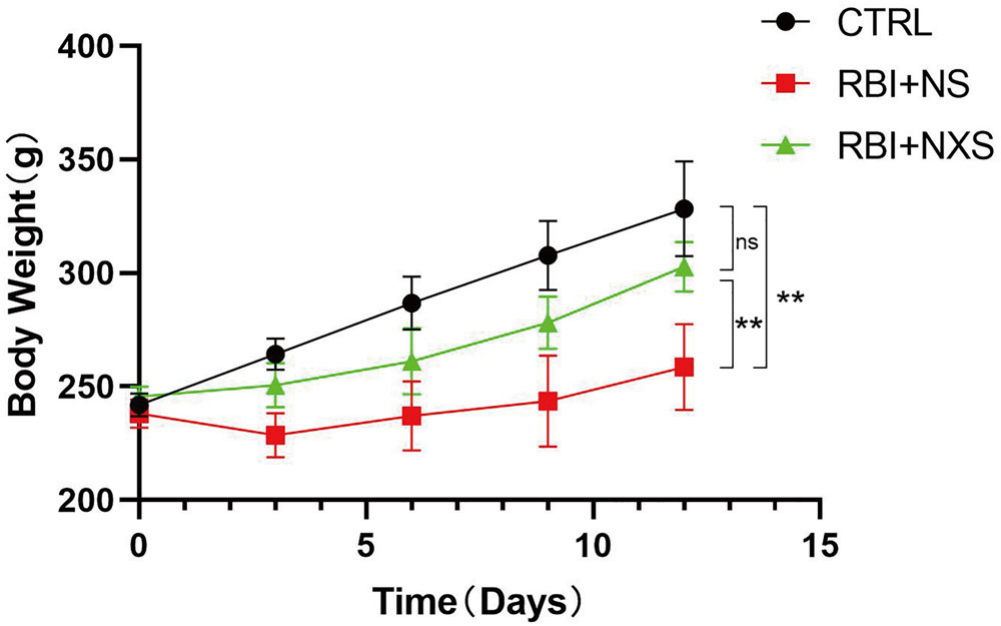
Body weight changes of rats in each group after irradiation and treatment. Body weight was monitored at the indicated time points in the Control (CTRL), radiation + normal saline (RBI+NS), and radiation + Naoxueshu Oral Liquid (RBI+NXS) groups. Data are presented as mean ± SD. According to Section 2.2, *n* = 6 rats per group were measured at each time point. Between‐group comparisons at each time point were performed using one‐way ANOVA with multiple‐comparison correction (Section 2.2), and significance at Day 12 is indicated on the graph (*p* < 0.01; ns, *p* > 0.05).

### 3.2 Effect of Naoxueshu Oral Liquid on Brain Water Content in Irradiated Rats

The measurement results of brain water content on Days 3, 7, and 14 after modeling (Figure 3A–C) revealed differences in brain water content among groups at the assessed time points. The irradiation + saline group maintained relatively high brain water content during the observation period, with values of (64.00 ± 0.82)% on Day 3 and (65.43 ± 0.95)% on Day 14. In contrast, the irradiation + Naoxueshu Oral Liquid group showed lower brain water content at the later assessed time points. The brain water content was (63.80 ± 0.64)% on Day 3, which was comparable to that of the irradiation + saline group (U = 17, *p* = 0.937). It was (63.10 ± 0.82)% on Day 7 and (63.73 ± 1.09)% on Day 14, remaining lower than the corresponding values in the irradiation + saline group and close to the level observed in the blank control group [(62.67 ± 0.94)%]. These findings suggest that Naoxueshu Oral Liquid was associated with attenuation of irradiation‐related brain water accumulation during the early post‐irradiation period.

**FIGURE 3:**
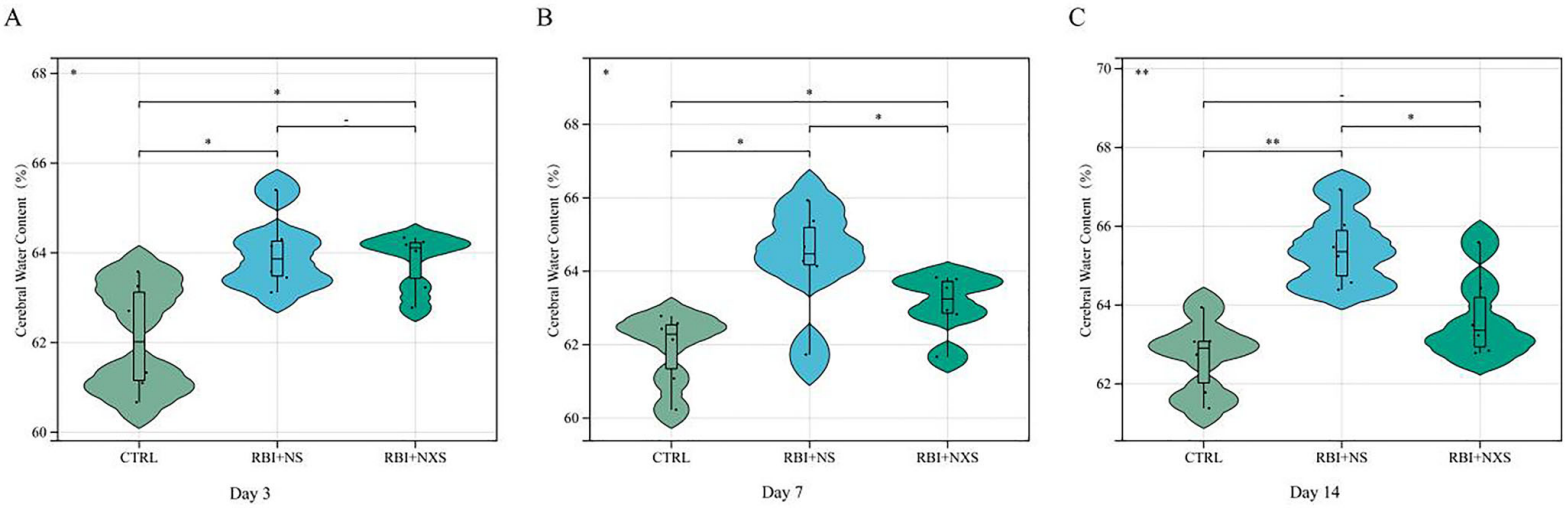
Changes in brain water content across groups at different time points after irradiation. Brain water content was assessed in CTRL, RBI+NS, and RBI+NXS groups at Day 3 (A), Day 7 (B), and Day 14 (C) after irradiation. Data are presented as mean ± SD. According to Section 2.2, *n* = 6 rats per group were analyzed at each time point. Intergroup comparisons were performed as described in Section 2.2. For brain water content, the Kruskal–Wallis test was first used for overall comparison among the three groups at each time point. If the overall test indicated a significant difference, pairwise comparisons were performed using Bonferroni‐corrected Mann–Whitney *U* tests. The significance markers shown in the figure correspond to the corrected pairwise comparisons. *p < 0.05; **p < 0.01; ns, *p* > 0.05. Each bracket denotes the specific pairwise comparison shown.

### 3.3 Effect of Naoxueshu Oral Liquid on Brain Histopathology in Irradiated Rats

HE staining results (Figure 4A–C) revealed the following morphological features: Representative images were obtained from the hippocampal dentate gyrus (DG) (Figure 4A) and the thalamic region (Figure 4B, C) at Day 7 and Day 14. Blank control group: Neurons exhibited clear nuclear staining, visible nucleoli, and uniformly distributed cytoplasm. The pericellular space appeared normal without significant edema. The perivascular space was unremarkable. After irradiation: Neuronal somata were swollen with pyknotic nuclei (some deeply stained), pale staining, and rounded morphology. The pericellular space was reduced. Marked widening of the perivascular space was observed, accompanied by vascular‐associated morphological alterations. After treatment with Naoxueshu Oral Liquid, the above pathological changes were alleviated, with reduced neuronal swelling and less prominent perivascular enlargement in the representative sections compared with the irradiation + saline group. Consistent with these morphological observations, the quantified PVS area fraction shown in Figure 4C provided semiquantitative support for perivascular space enlargement after irradiation. PVS area fraction was significantly increased in the irradiation + saline group compared with the blank control group at Day 7 (*p* < 0.001) and Day 14 (*p* < 0.01). Compared with the irradiation + saline group, Naoxueshu treatment significantly reduced PVS area fraction at both Day 7 and Day 14 (both *p* < 0.01). These findings support that Naoxueshu treatment attenuated irradiation‐associated perivascular space enlargement.

**FIGURE 4:**
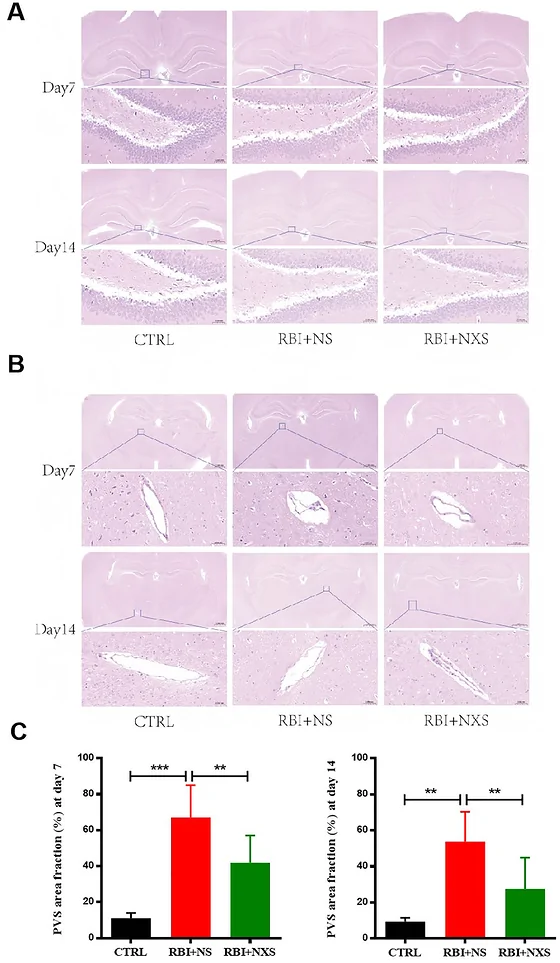
Histopathological changes in brain tissue after irradiation and Naoxueshu treatment (H&E staining). Representative hematoxylin and eosin (H&E)‐stained coronal brain sections from CTRL, RBI+NS, and RBI+NXS groups at Day 7 and Day 14 after irradiation. (A) Representative images from the hippocampal dentate gyrus (DG) showing cellular morphological changes. (B) Representative images from the thalamic region showing vascular/perivascular morphological changes (including perivascular space alterations). (C) PVS area fraction among groups. Images were acquired at comparable anatomical levels using a consistent imaging protocol across animals. According to Section 2.2, *n* = 6 rats per group were analyzed at each time point; representative images are shown. Scale bar = 50 µm.

### 3.4 Effect of Naoxueshu Oral Liquid on Morris Water Maze Performance in Irradiated Rats

Morris water maze test results (Figure 5A–C) showed that in the probe trial performed on Day 14 post‐irradiation, the trajectory patterns of rats in each group were as follows (Figure 5A): Blank control group: Trajectories were predominantly concentrated in the target quadrant, with direct paths and minimal circling. Irradiation + saline group: Paths were scattered and disorganized, with extensive circling in non‐target quadrants and prolonged search time. Irradiation + Naoxueshu Oral Liquid group: Trajectories resembled those of the blank group, largely focused near the target quadrant with reduced meandering. In terms of latency to the first crossing of the former platform location (Figure 5B): The irradiation + saline group showed a significantly prolonged latency to the first crossing of the former platform location (43.33 ± 4.63 s) compared to the blank group (33.50 ± 1.87 s, *p* < 0.01). The irradiation + Naoxueshu group exhibited markedly shortened latency (35.67 ± 1.51 s), which was not significantly different from the blank group (*p* = 0.232) but significantly shorter than the irradiation + saline group (*p* < 0.01). Regarding the number of platform crossings within 60 s (Figure 5C): The irradiation + saline group crossed the platform site significantly less frequently (2.5 ± 0.55 times) than the blank group (5.17 ± 1.7 times, *p* < 0.01). The irradiation + Naoxueshu group showed increased crossings (4 ± 0.63 times), which did not differ significantly from the blank group (*p* = 0.07) but was significantly higher than the irradiation + saline group (*p* < 0.01). These findings indicate improved Morris water maze task performance after Naoxueshu treatment, as reflected by shorter latency and increased platform crossings in the probe trial.

**FIGURE 5:**
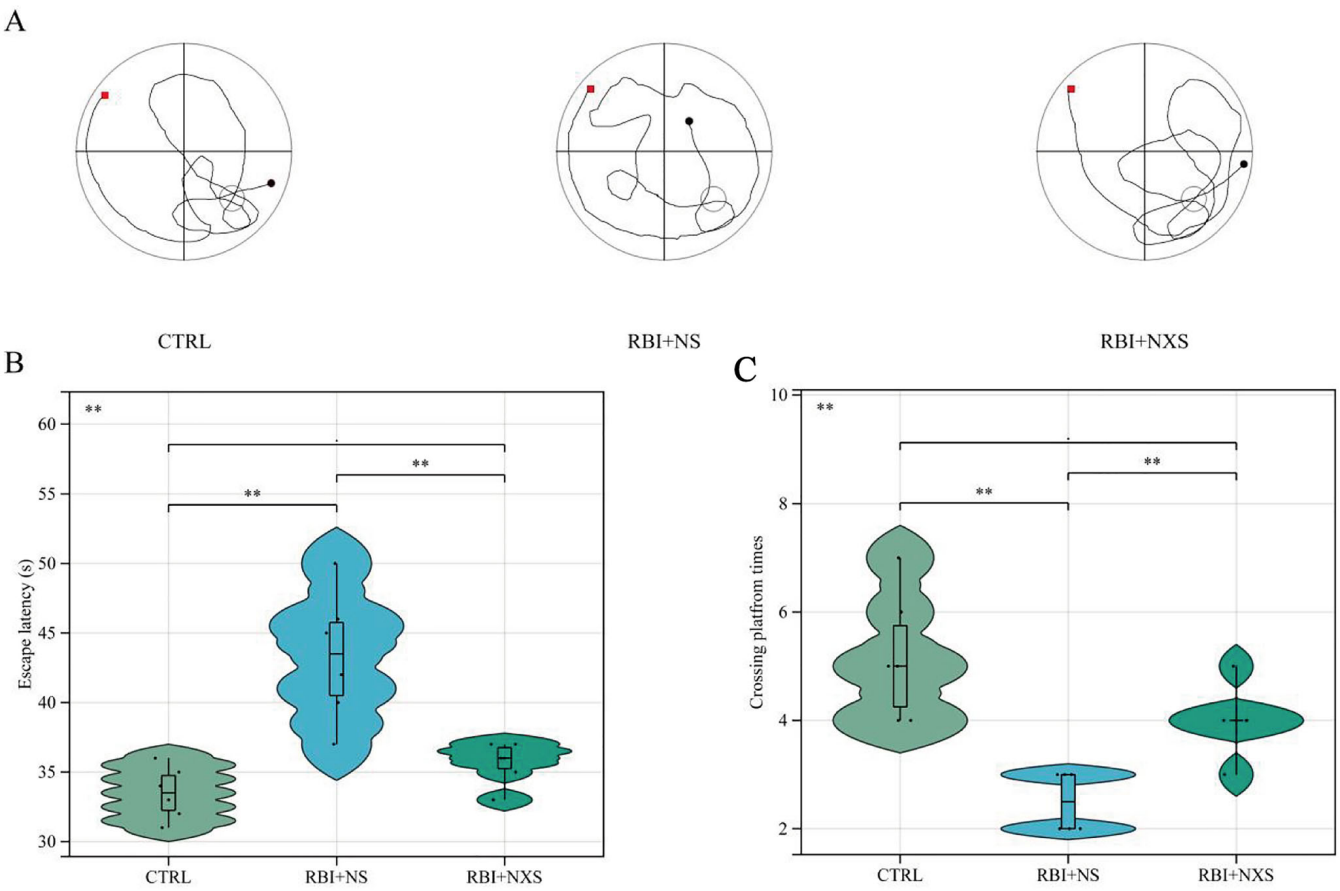
Morris water maze performance after irradiation and Naoxueshu treatment. (A) Representative swim trajectories for each group (red markers indicate starting points; black markers indicate endpoints). (B) Latency to first reach/cross the former platform location and (C) number of platform crossings within 60 s during the probe trial. Rats assigned to the Morris water maze cohort were identified on Day 8 post‐irradiation, trained from Day 8 to Day 13 post‐irradiation, and tested in the probe trial on Day 14 post‐irradiation. According to Section 2.2, *n* = 6 rats per group were included for Morris water maze analysis. Data are presented as mean ± SD. Between‐group comparisons were analyzed as described in Section 2.2 (one‐way ANOVA), and significance is indicated on the brackets. *p < 0.05; **p < 0.01; ns, *p* > 0.05. Each bracket denotes the specific pairwise comparison shown.

## 4 Discussion

Radiation‐induced brain injury is a common complication following radiotherapy for head and neck malignancies. Its core pathological mechanisms involve multiple processes, including inflammatory response, oxidative stress, blood–brain barrier disruption, and neuronal apoptosis, ultimately leading to symptoms such as cerebral edema and cognitive decline (Brunaud et al. 2024; Cao 2019; Gorbunov and Kiang 2021; Gottschalk et al. 2021; Jin et al. 2021; Li and Lan 2011; Li et al. 2023; Liu et al. 2023; Luo et al. 2015; Morris 1984; Pazzaglia et al. 2020; Shandong Wolwa Pharmaceutical Technology Co., Ltd 2008; Shi and Ma 2024; Song et al. 2022; Wang et al. 2024; Wei et al. 2020; Yao et al. 2023). Currently, there is a lack of specific clinical treatments for this condition. Traditional Chinese medicine, with its multi‐target regulatory advantages, has shown potential in neural injury repair. This study showed that Naoxueshu Oral Liquid was associated with better body‐weight recovery, lower brain water content, less severe histopathological changes, and better performance on the measured Morris water maze endpoints in rats with radiation‐induced brain injury. Because no biochemical or molecular endpoints were collected, mechanistic interpretation of these effects is limited, and the following discussion is framed as literature‐supported hypotheses rather than direct experimental evidence.

Naoxueshu Oral Liquid is composed of seven herbal ingredients, including *Astragalus membranaceus* (Huangqi), *Hirudo* (Shuizhi), *Acorus tatarinowii* (Shichangpu), *Cyathula officinalis* (Niuxi), *Cortex moutan* (Mudanpi), *Rheum palmatum* (Dahuang), and *Ligusticum chuanxiong* (Chuanxiong) (Wang et al. 2024). Among these, *Astragalus* and Chuanxiong are commonly considered key components in neurovascular protection in prior studies. In the experiment, irradiated rats exhibited slowed weight gain; however, following treatment with Naoxueshu Oral Liquid, their weight gradually recovered. This phenotypic improvement may be consistent with reduced systemic stress and/or neuroinflammatory burden, but inflammatory readouts were not measured and therefore this cannot be concluded from the present data. *Astragalus* total flavonoids have been shown to inhibit inflammatory signaling pathways such as NF‐κB and p38 MAPK, reducing microglial activation and the release of pro‐inflammatory cytokines including IL‐1β and TNF‐α (Sun and Wang 2024; Zhang et al. 2025). Radiation‐induced neuroinflammation is a key factor leading to dysfunction of the hypothalamic feeding center and subsequent weight loss (Chaves et al. 2020). Additionally, *Astragalus* has been reported to activate the PI3K/Akt pathway and reduce neuronal apoptosis (Tan et al. 2020; Zhang et al. 2025). These findings provide a literature‐based rationale for potential neuroprotective effects, but they do not establish that this pathway mediated the weight recovery observed in the present study.

In terms of ameliorating cerebral edema, this study found that after 7 and 14 days of continuous administration of Naoxueshu Oral Liquid, the brain water content significantly decreased, approaching normal levels by Day 14. Because BBB integrity was not directly assessed in this study (e.g., hippocampal parenchymal IgG/fibrinogen extravasation, or tight‐junction proteins such as ZO‐1), BBB disruption and a BBB‐protective mechanism cannot be concluded from the current dataset. Nevertheless, existing studies suggest that components of Chuanxiong may enhance antioxidant capacity and protect vascular endothelial function (Li et al. 2019; Marco‐Contelles 2020; Niu et al. 2022; Yu et al. 2018). Prior reports also indicate potential regulation of tight junction proteins (e.g., ZO‐1) and BBB permeability in other injury models (Chen et al. 2023; Tan et al. 2015; Wang et al. 2024; Wang et al. 2019); whether Naoxueshu exerts similar effects in this RBI model requires direct BBB‐focused validation. The observed time‐dependent improvement starting from Day 7 may be biologically compatible with previous reports on ligustrazine‐related vascular and microcirculatory effects; however, endothelial repair was not directly assessed in the present study.

Histopathological examination of brain tissue revealed that Naoxueshu Oral Liquid ameliorated post‐irradiation damage, including ill‐defined neural structures and vascular‐associated morphological alterations (Chen et al. 2017; Guo et al. 2017). In addition to descriptive H&E observations, the quantified PVS area fraction shown in Figure 4C provided semiquantitative support for irradiation‐associated perivascular space enlargement and its attenuation after Naoxueshu treatment. This finding further supports the interpretation that Naoxueshu may alleviate vascular‐associated histological alterations in the early phase after irradiation. From a mechanistic perspective, *Astragalus* has been reported to modulate neuroinflammation and reduce cellular edema in related contexts, while ligustrazine has been linked to endothelial protection and microcirculatory support (Cao et al. 2020). Further studies using dedicated cellular and molecular markers are warranted to clarify the mechanisms underlying these morphological improvements.

Another finding was the improvement in Morris water maze performance. In the present study, cognitive‐related outcomes were assessed only using the Morris water maze. Naoxueshu Oral Liquid shortened the latency to the first crossing of the former platform location in irradiated rats and increased the number of platform crossings, indicating improved spatial learning and memory performance in this task. Together, these results support an improvement in Morris water maze task performance after Naoxueshu treatment. Because the behavioral assessment in this study was based on latency and platform crossings, without measurements of swimming speed or total distance, the interpretation was restricted to the measured Morris water maze endpoints rather than generalized to overall neurocognitive protection. Additional movement‐related parameters may provide complementary information in future behavioral evaluations. From a biological perspective, the hippocampus is a key region involved in spatial learning and memory and is particularly sensitive to irradiation (Ding et al. 2022; Franco‐Pérez et al. 2020; Wu et al. 2022). Although hippocampal neuronal protection was not directly assessed in the present study, previous evidence provides a plausible basis for considering related pathways in the interpretation of the behavioral findings. *Astragalus* total flavonoids have been reported to modulate MAPK signaling and reduce apoptosis‐related processes (Xiang et al. 2025; Xiao et al. 2025), and ligustrazine has been associated with reduced caspase‐3 activation in other models (Hu et al. 2023; Li et al. 2023). These findings support possible links between Naoxueshu‐related components and neuronal stress‐response pathways; however, such mechanisms remain to be verified directly in the present RBI model. Key processes relevant to post‐irradiation cognitive dysfunction, including hippocampal apoptosis, neuroinflammation, BBB leakage, oxidative/nitrosative damage, and neurogenesis, were not evaluated directly in this study. In particular, hippocampal neurogenesis, commonly assessed using markers such as DCX and Ki67, represents an important mechanistic endpoint for future investigation. The timing of behavioral assessment should also be considered when interpreting the present findings. Compared with studies that describe radiation‐induced neurocognitive dysfunction as a process that may evolve over longer post‐irradiation periods or emphasize neurovascular injury patterns after ionizing irradiation, the present experiment was designed to capture early pathological and behavioral changes within a 14‐day acute high‐dose model (Gorbunov and Kiang 2021; Rübe et al. 2023). Behavioral assessment in the current study was performed at 14 days post‐irradiation, whereas Sun et al. (2016) reported modest MWM impairment at 1 month after 30 Gy using a larger cohort. Together, these studies indicate that 30‐Gy whole‐brain irradiation can be used to examine radiation‐associated behavioral changes across different post‐irradiation windows, while the present study specifically focused on the early 14‐day phase. Differences in follow‐up duration, behavioral endpoints, and experimental procedures may therefore contribute to variations in the magnitude and pattern of cognitive‐related changes observed across studies. In addition, early behavioral improvement may reflect a combination of treatment‐associated effects on irradiated brain tissue and broader systemic recovery during the post‐irradiation period. Longer‐term studies will be useful for determining whether the observed benefits persist as radiation‐induced brain injury evolves over time.

This study has certain limitations. We did not thoroughly investigate the molecular mechanisms by which Naoxueshu modulates inflammatory factors (e.g., IL‐1β, TNF‐α), oxidative stress markers (e.g., ROS, SOD), or tight junction proteins (e.g., ZO‐1), nor did we directly demonstrate the interaction between drug components and these pathways. Future studies should incorporate biochemical and molecular measurements (e.g., cytokines, 3‐NT/8‐OHdG, apoptosis markers, and BBB integrity indices) to validate the proposed mechanisms and link them to the observed phenotypes. In addition, BBB integrity was not evaluated in this work (e.g., IgG/fibrinogen parenchymal infiltration in the hippocampus), so BBB leakage and Naoxueshu‐mediated BBB protection remain to be confirmed in future studies. Hippocampal neurogenesis was also not assessed; future studies will include DCX (immature neuron marker) and Ki67 (proliferation marker) staining to evaluate neurogenesis‐related changes after irradiation and their potential modulation by Naoxueshu. Second, the irradiation paradigm in this study (single‐dose 30 Gy whole‐brain irradiation) differs from clinical radiotherapy, which is typically delivered using fractionated regimens. Therefore, the 14‐day behavioral and pathological findings should be interpreted as early responses within an acute high‐dose model rather than as evidence of the long‐term evolution of clinical radiation‐induced brain injury. The present findings should therefore be interpreted within the context of an acute experimental RBI model. Future studies incorporating clinically relevant fractionated irradiation schedules and longer observation periods will be important for extending these findings toward translational evaluation. Accordingly, these findings should not be extrapolated directly to clinical efficacy, dose equivalence, safety, long‐term neurocognitive benefit, chronic radiation‐induced brain injury, or human treatment response. Finally, although each group initially contained 36 rats, animals were pre‐allocated to different terminal endpoints at specific time points (Days 3/7/14 for wet‐dry measurements; Days 7/14 for histology; and Day 14 for MWM), resulting in an effective *n* = 6 per group for each analysis. Because no a priori power calculation was performed, this endpoint‐specific sample size should be considered exploratory and may have limited statistical power for detecting smaller effects. Larger cohorts with prospective sample size estimation will be valuable for future validation studies. In addition, because only male rats were included, the findings may not fully generalize to females; future studies should incorporate both sexes to evaluate potential sex‐specific responses.

## 5 Conclusion

In this exploratory study using a single‐dose (30 Gy) whole‐brain irradiation rat model, Naoxueshu Oral Liquid attenuated early radiation‐induced brain injury, as evidenced by reduced brain water content, alleviated histopathological alterations, and improved performance on the measured Morris water maze endpoints compared with saline‐treated irradiated rats. The consistency of these effects across physiological, histopathological, and behavioral endpoints suggests that Naoxueshu Oral Liquid may have a protective effect during the early stage of radiation‐induced brain injury. Collectively, these findings provide preliminary preclinical evidence for the potential therapeutic value of Naoxueshu Oral Liquid in early radiation‐induced brain injury and support its further evaluation in adequately powered studies, together with subsequent investigation of the underlying mechanisms and translational applicability.

## Author Contributions

Conception and design of the work: **Fuguang Li**. Data collection: **Xiaolong Qu**, **Kuiyuan Li**, **Guosong Li**, **Shifang Zhou**, **Min Zhao**, **Chunyu Zhang**, **Lei Hui**, **Wenchao Li**. Analysis and interpretation of the data: Xiaolong Qu, Kuiyuan Li, Guosong Li, Shifang Zhou, Min Zhao, Chunyu Zhang, Lei Hui, Wenchao Li. Statistical analysis: Fuguang Li, Lei Hui, Wenchao Li. Supervision: Fuguang Li. Drafting the manuscript: Fuguang Li. Critical revision of the manuscript: All authors. Approval of the final manuscript: All authors.

## Funding

This study is supported by Medical Science and Technology Research and Development Program of Henan Province in 2024 (NO: LHGJ20240486).

## Ethics Statement

The experimental protocol was approved by the Animal Experimentation Ethics Committee of The First Affiliated Hospital of Henan Medical University (Approval No.: EC‐023‐217).This study was conducted in accordance with the principles on ethical animal research outlined in the Basel Declaration and the ethical guidelines by the International Council for Laboratory Animal Science (ICLAS). All experimental protocols were conducted following the ARRIVE guidelines.

## Conflicts of Interest

The authors declare no conflicts of interest.

## Data Availability

All data generated or analyzed during this study are included in the article.
